# The Value of Zoological Gardens to Education

**Published:** 1889-01

**Authors:** 


### EDITORIAL DEPARTMENT.

### THE VALUE OF ZOOLOGICAL GARDENS TO EDUCATION.

IT is believed by the friends of the bill having for its object the establishment of a National Zoological Garden at Washington, that its failure to pass at the last session of Congress, was due more to neglect than to actual disinclination to further the scheme. It is certainly to be hoped that such is the case, and after all, considering the exigencies of a situation which urged the legislator in our national halls to devote the major portion of his time during the session to those other zoological questions—the Canadian Fisheries Treaty and the bill for the exclusion from our shores, of the almond-eyed product of the Celestial Kingdom, he may perhaps be pardoned for feeling that the claims of Natural History were fully satisfied. But the closing of the late political campaign has now retired these engrossing subjects from his consideration, a new session has begun, and the measure should be promptly revived, pushed to enactment, and the enterprise begun with as little delay as possible.

The project originated long since, with the late Secretary of the Smithsonian Institution, Professor Baird, and having in view the peerless liberality of the general government in the prosecution of scientific work, and the splendid resulting series of publications which have followed one another from the government printing office, we can not but regard it as strange that attention has not been more strongly drawn to the need of multiplying just such institutions as this—invaluable as adjuncts to observation, and as store-houses of material for exact work in more than one branch of natural science.

The fact that under modern scientific methods, the objective study of man, on lines of research tending to results of high importance—the most important of all, in regard to his needs and welfare—is largely based upon similar observation of the lower animals, receives fresh demonstration with almost every advance in biology—and it is in collections of living animals that the field for such investigations is largely found. The human anatomist, the human physiologist, pathologist and even psychologist of to-day, save for the astonishing development, within a few years, of the comparative side of those branches of study, would stand about where his grandfather did, in prospecting the paths of science.

And partly aside from the scientific, the popular value of exhibitions of living wild animals, deserve more serious consideration than it generally receives in the United States.

Just when the more or less universal interest which man feels in the birds and beasts which surround him in nature, originated, we cannot say—perhaps a due sense of ancestral pride made it coincident with his first dawnings of self-consciousness—but it certainly must have grown with his pursuit of the chase and strengthened with his early attempts at the subjugation of useful animals and their keeping for domestic purposes. It was surely among his earliest instincts.

The desire of conquering potentates, in later years, to magnify the pomp of triumphal processions by the exhibition of strange and fearful animal forms from foreign lands, probably led to the formation of such collections as that which it is said Darius located at Persepolis, but it was two thousand years before Louis XIV, with the aid of Buffon—founded the *Jardin du Roi*, from which the present *Jardin des Plantes* is descended—that Alexander of Macedon, made the first scientific zoological collection for Aristotle, the first zoologist. For the past hundred years the importance of such collections, as means of public education has been so universally recognized in civilized countries, that the fact no longer needs demonstration. In Europe, hardly a city of the first or even second class is without a zoological exhibit of greater or less comprehensiveness, many of them owing their means of existence to government subsidy, others under private management, but kept up solely for the public good. Those of London, Paris, Antwerp, Amsterdam, Frankfort and Berlin are known throughout the world. Even India and Australia have extensive and prosperous gardens.

In our own country, for reasons of which we are from time to time reminded by the more intelligent of our foreign critics—the late Matthew Arnold, for example—but which are indignantly repudiated by the average citizen of the Republic, neither popular science, nor popular art, nor popular any thing which leads elsewhere than to material advantage, at present receives much encouragement from the mass of population, and zoological gardens have been successfully established in few of our cities. In Woodward's garden, at San Francisco, there are a few animals. St. Louis and Cincinnati have gardens which at their outset promised fairly, but whose present condition does not indicate a high degree of prosperity.

Chicago has the nucleus of a public collection, mostly of native species, in Lincoln Park. In New York, the collection at Central Park is supported by appropriations from the city, but is cramped in space, and has only lately begun to receive, in some measure, the support merited by the intelligent energy which had previously kept it alive. The Zoological Society of Philadelphia modeled itself and its garden largely upon that in London, and was the pioneer in America, but though its facilities and its collection rank with the best in Europe, its support from public admissions is precarious and it undergoes periodic struggles with poverty.

Popular zoology is thus in its infancy, on this side of the Atlantic, and each and every one of the institutions named, needs substantial encouragement on the road to final success. Our museums of dead, stuffed and inorganic material receive frequent endowments and donations, but up to the present time, at least, the necessities of living collections do not seem to have appealed forcibly to donors and testators. It is difficult to say just why this is so, but the fact leaves only the alternatives of maintaining them by governmental aid or by charging an admission fee. When the latter means is adopted, the object can be obtained only by building up a considerable degree of public interest in the subjects dealt with, and it can hardly be doubted that the establishment of a suitable zoological garden under government patronage at the National Capitol would give a stimulus to interest, not only there, but throughout the country—greatly beneficial to education in popular zoology, and this in turn would certainly lead to desires and attainments far beyond its own immediate sphere of influence.

From another aspect—there has been enough of neglect in the relation of our government to the aborigines of all classes. From the day of the advent of the white man, the trails of the Indian have converged towards one point—extinction; our bison grandest of the bovine race—has steadily followed the route, along which the *Rhytina* had preceded him; the elk—noblest of the deer tribe—is already far advanced in the same path; the beautiful parrot, which was the only representative of its group in North America, has now almost joined the Dodo, among the things that were, and so on through the list.

Much of this is, of course, inseparable from the developing needs and arts of civilization, but on the other hand, a great deal of the resulting evil may be avoided by the timely application of the very means which have led to it. The age of the hunter is fast passing into the age of the student, and what remains to us from the pursuit of the former, civilization must now preserve to supply material for the researches of the latter. Zoological gardens and protected parks must take the place of native ranges to all of our species which it is possible to keep artificially—the only surety of whose preservation is in the multiplication of such institutions either by private generosity, or by public allowance in every large city, and by according a more liberal support to those already existing. B.

